# Supplementary material for: Learning future terrorist targets through temporal meta-graphs
Source: Sci Rep. 2021 Apr 20;11:8533. doi: 10.1038/s41598-021-87709-7 (PMC8058089; doi:10.1038/s41598-021-87709-7)
Supplement: Supplementary file 1 — Supplementary Information 1. [file 41598_2021_87709_MOESM1_ESM.pdf]

Learning Future Terrorist Targets Through Temporal Meta-Graphs: SUPPLEMENTARY MATERIALS

Gian Maria Campedelli[‡,1] Mihovil Bartulovic[2] Kathleen M. Carley[2]

[‡] Corresponding Author: gianmaria.campedelli@unitn.it

[1] Department of Sociology and Social Research - University of Trento (Italy)

[2] School of Computer Science - Carnegie Mellon University (United States)

Abstract

In the last twenty years, terrorism has led to hundreds of thousands of deaths and massive economic, political, and humanitarian crises in several regions of the world. Using real-world data on attacks occurred in Afghanistan and Iraq from 2001 to 2018, we propose the use of temporal meta-graphs and deep learning to forecast future terrorist targets. Focusing on three event dimensions, i.e., employed weapons, deployed tactics and chosen targets, meta-graphs map the connections among temporally close attacks, capturing their operational similarities and dependencies. From these temporal meta-graphs, we derive two-day-based time series that measure the centrality of each feature within each dimension over time. Formulating the problem in the context of the strategic behavior of terrorist actors, these multivariate temporal sequences are then utilized to learn what target types are at the highest risk of being chosen. The paper makes two contributions. First, it demonstrates that engineering the feature space via temporal meta-graphs produces richer knowledge than shallow time-series that only rely on frequency of feature occurrences. Second, the performed experiments reveal that Bi-directional LSTM networks achieve superior forecasting performance compared to other algorithms, calling for future research aiming at fully discovering the potential of artificial intelligence to counter terrorist dynamics.

S1 Datasets: Descriptive Statistics

S1.1 Preliminary Filtering

According to the Global Terrorism Database (GTD) (LaFree and Dugan, 2007), between 2000 and 2018, Afghanistan and Iraq recorded a total of 14,385 and 25,896 terrorist attacks, respectively. As described in the main manuscript, two levels of criteria are imposed for the inclusion of an event in the GTD. Besides these two levels, an additional variable is added to the dataset mapping those events for which doubt exist regarding their terrorist nature.

In order to avoid biases and noise in our signals, during the generation of the proposed meta-graphs and the time series, we proceeded to exclude all those events that were doubtful in terrorist nature, according to the *doubtterr* variable. This led to a slight reduction in the total number of attacks: 12,120 attacks for Afghanistan and 22,773 for Iraq.

The next two subsections will provide an overview of the descriptive statistics of the temporal meta-graph derived multivariate time series for both Afghanistan and Iraq.

S1.2 Afghanistan

The Afghanistan multivariate time-series, derived from our framework, focus on two-day time units, for a total of 3,289 data points (the same for Iraq). In the period under consideration, 939 units did not record attacks, a 28.54% of the total. The histogram below (Figure S1) reports the count of non-zero features in the considered time series, for each time unit (i,e., how many weapons, tactics and targets have a centrality value higher than 0 at time unit u?).

S1.2.1 Tactics

Terrorist actors that were active in Afghanistan between 2000-2018 have exploited 9 different tactic types: *Unknown, Bombing/Explosion, Hijacking, Armed Assault, Facility/Infrastructure Attack, Assassination, Hostage Taking (Kidnapping), Unarmed Assault, Hostage Taking (Barricade Incident)*. Below are reported the number of occurrences of each tactic type (Table 1).

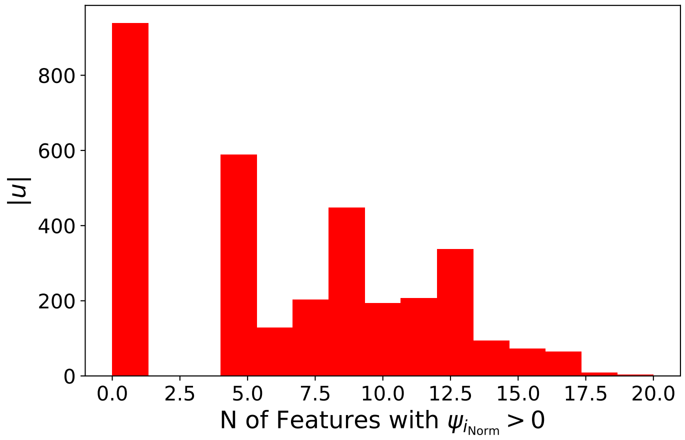

Figure S1: Count of non-zero $\psi_{i_{\text{Norm}}}[u]$ in each u - Afghanistan.The figure has been created using Matplotlib version 3.1.3. Url: https://matplotlib.org/3.1.3/contents.html.

**Tactic** x_i	**N of** $\psi_{i_{\text{Norm}}}[u] > 0$	**%**
Bombing/Explosion	1961	59.62%
Armed Assault	1442	43.84%
Hostage Taking (Kidnapping)	808	24.57%
Assassination	781	23.75%
Unknown	606	18.43%
Facility/Infrastructure Attack	335	10.19%
Unarmed Assault	59	1.79%
Hostage Taking (Barricade Incident)	36	1.09%
Hijacking	18	0.55%

Table 1: Afghanistan Tactics - Number of non-zero $\psi_{i_{\text{Norm}}}[u]$ occurrences and percentage over U

All tactics are kept in the experimental analyses. Figure S2 showcases the distribution of each tactic in terms of centrality values.

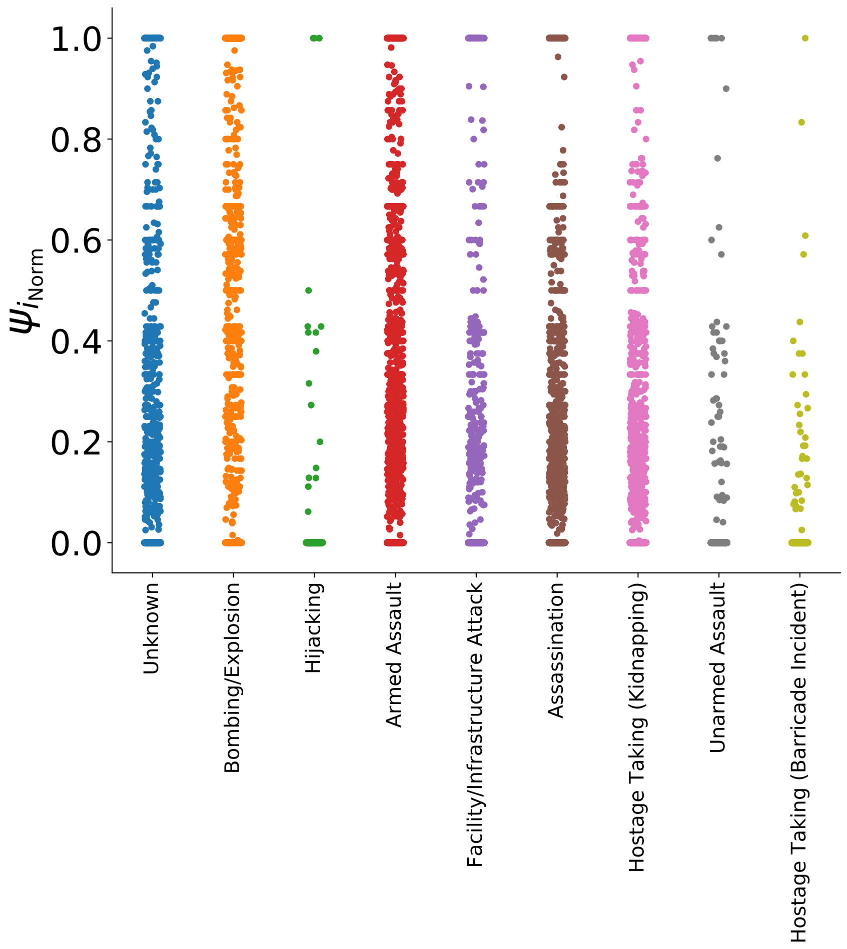

Figure S2: Distribution of $\psi_{i_{\text{Norm}}}[u]$ over U for all the tactics features X - Afghanistan. The figure has been created using Matplotlib version 3.1.3. Url: https://matplotlib.org/3.1.3/contents.html.

S1.2.2 Weapons

A total of 7 weapon categories were utilized by terrorists in Afghanistan, these are: *Unknown Explosives, Firearms, Incendiary, Melee, Sabotage Equipment, Other.* Below we report the distribution of these weapons (Table 2).

**Weapon** x_j	**N of** $\psi_{j_{\text{Norm}}}[u] > 0$	**%**
Explosives	2020	61.42%
Firearms	1624	49.38%
Unknown.1	876	26.63%
Incendiary	321	9.76%
Melee	194	5.90%
Other	6	0.18%
Sabotage Equipment	4	0.12%

Table 2: Afghanistan Weapons - Number of non-zero $\psi_{j_{\text{Norm}}}[u]$ occurrences and percentage over U

Other and *Sabotage Equipment* have been filtered out from the final set of weapons given their extremely low prevalence, leading to a total of 5 weapons. The centrality value distributions of the features in the final set of weapons X are shown in Figure S3.

S1.2.3 Targets

In the 2000-2018 period, a total of 21 target categories were hit at least once by terrorists: *Terrorists/Non-State Militia, Government (General), Private Citizens & Property, Airports & Aircraft, Unknown Government, (Diplomatic) Journalists & Media, Police, Business, Religious Figures/Institutions, Military, Educational Institution, Telecommunication, Transportation, NGO, Food or Water Supply, Tourists, Utilities, Other, Violent Political Party, Maritime.* Below Table 3 shows the distribution of these targets.

Given the low prevalence, as done for all the other dimensions (in both datasets) we have proceeded to exclude those features that were present less than 10 times of the course of the entire 2000-2018 period. *Tourists* and *Maritime* are thus excluded from the experiments, leading to a total of 18 targets (Figure S4).

S1.3 Iraq

In the Iraq case, out of the 3,289 time units, 698 recorded no attacks (21.2%), thus having all the associated time series with 0 as inputs. Below, the histogram reporting the count of non-zero features for all the multivariate time-series at each time unit u (Figure S5).

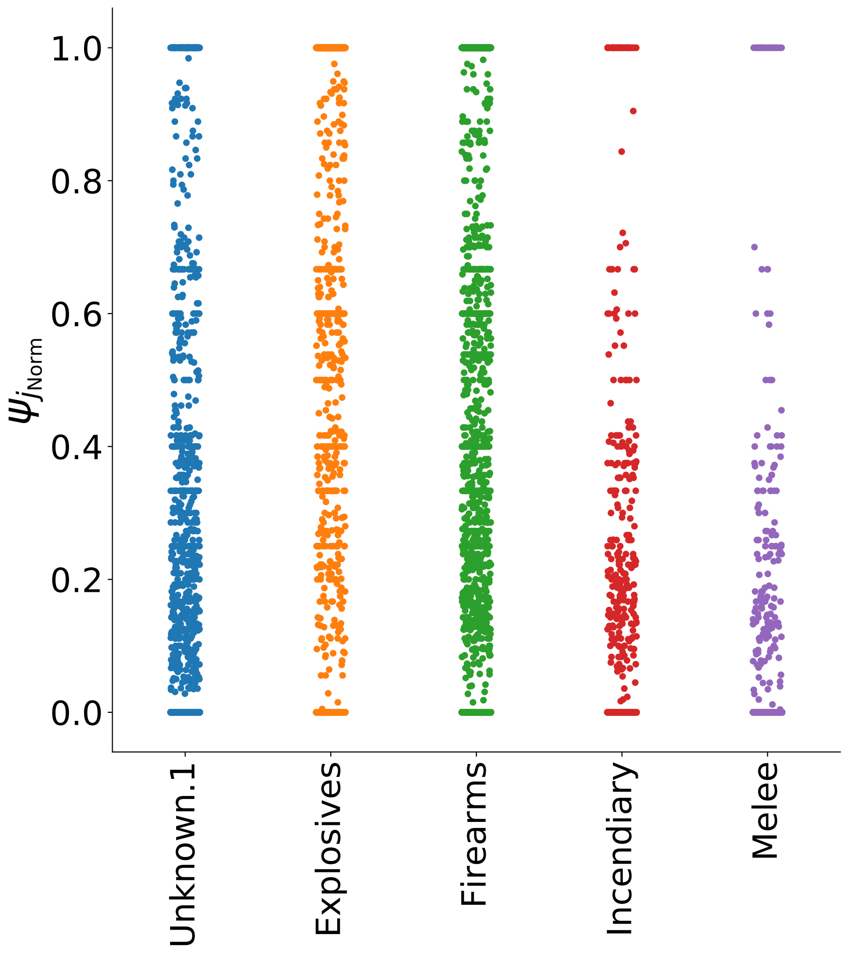

Figure S3: Distribution of $\psi_{j_{\text{Norm}}}[u]$ over U for all the weapons features W - Afghanistan. The figure has been created using Matplotlib version 3.1.3. Url: https://matplotlib.org/3.1.3/contents.html.

S1.3.1 Tactics

In Iraq, terrorists have employed 9 distinct tactics from 2000 to 2018: *Assassination, Bombing/Explosion, Unknown Armed Assault, Facility/Infrastructure Attack, Hostage Taking (Kidnapping), Unarmed Assault, Hostage Taking (Barricade Incident), Hijacking* . The distribution is reported below (Table 4).

Hijacking has been excluded from the multivariate time-series given the extremely low prevalence over the entire history U. The distribution of the values of features in X for Iraq are displayed below (Figure S6).

**Target** y_k	**N of** $\psi_{k_{\text{Norm}}}[u] > 0$	**%**
Private Citizens & Property	1635	49.71%
Police	1506	45.79%
Government (General)	1151	35.00%
Military	518	15.75%
Unknown.2	505	15.35%
Business	465	14.14%
Educational Institution	316	9.61%
Religious Figures/Institutions	231	7.02%
NGO	155	4.71%
Terrorists/Non-State Militia	154	4.68%
Transportation	144	4.38%
Government (Diplomatic)	133	4.04%
Journalists & Media	82	2.49%
Airports & Aircraft	54	1.64%
Telecommunication	53	1.61%
Utilities	38	1.16%
Violent Political Party	21	0.64%
Food or Water Supply	15	0.46%
Tourists	4	0.12%
Maritime	1	0.03%

Table 3: Afghanistan Targets - Number of non-zero $\psi_{k_{\text{Norm}}}[u]$ occurrences and percentage over U

**Tactic** x_i	**N of** $\psi_{i_{\text{Norm}}}[u] > 0$	**%**
Bombing/Explosion	2459	74.76%
Armed Assault	1440	43.78%
Assassination	863	26.24%
Hostage Taking (Kidnapping)	612	18.61%
Unknown	394	11.98%
Facility/Infrastructure Attack	136	4.13%
Hostage Taking (Barricade Incident)	25	0.76%
Unarmed Assault	10	0.30%
Hijacking	7	0.21%

Table 4: Iraq Tactics - Number of non-zero $\psi_{i_{\text{Norm}}}[u]$ occurrences and percentage over U

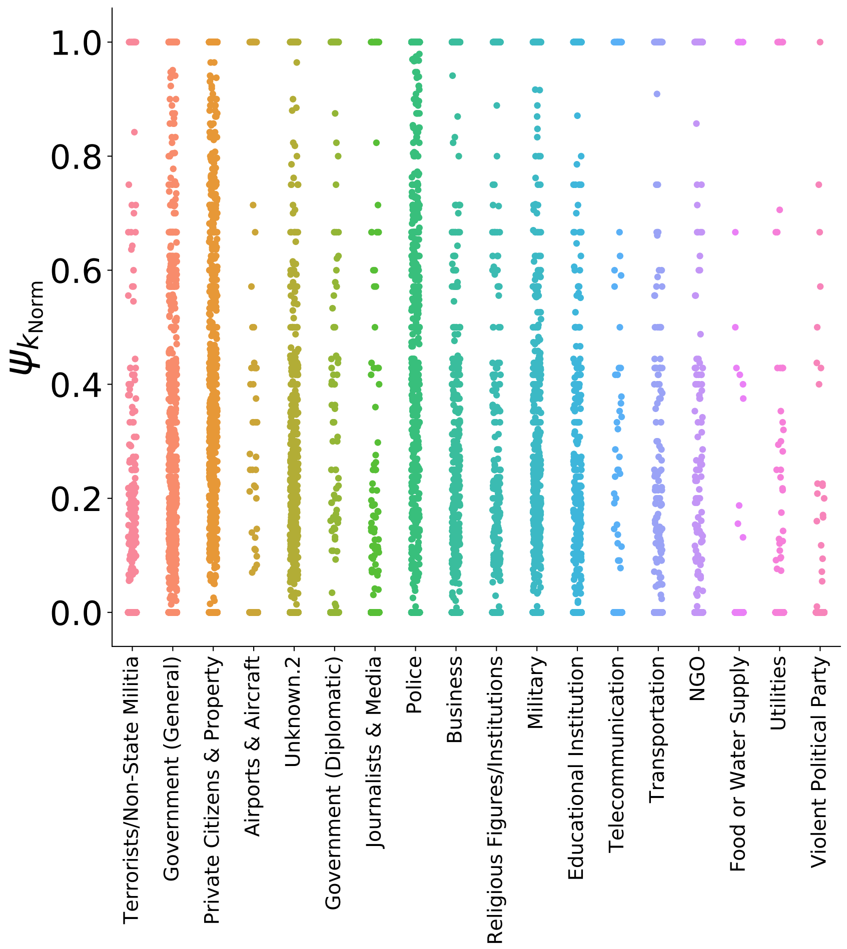

Figure S4: Distribution of $\psi_{k_{\mathrm{Norm}}}[u]$ over U for all the target features Y - Afghanistan. The figure has been created using Matplotlib version 3.1.3. Url: https://matplotlib.org/3.1.3/contents.html.

S1.3.2 Weapons

Overall, 7 weapon categories have at least one occurrence in the Iraq dataset: *Explosives, Firearms, Unknown, Incendiary, Melee, Other, Sabotage Equipment* (Table 5).

As done for the Afghanistan dataset, also in this case *Other* and *Sabotage Equipment* have been removed from the set of multivariate time series. The final set of W for Iraq is displayed in Figure S7.

S1.3.3 Targets

Overall, in Iraq 21 target types have been hit from 2000 to 2018. These are: *Private Citizens & Property, Government (Diplomatic), Business, Police, Government (General), NGO, Journalists & Media, Violent Political Party, Religious Figures/Institutions,*

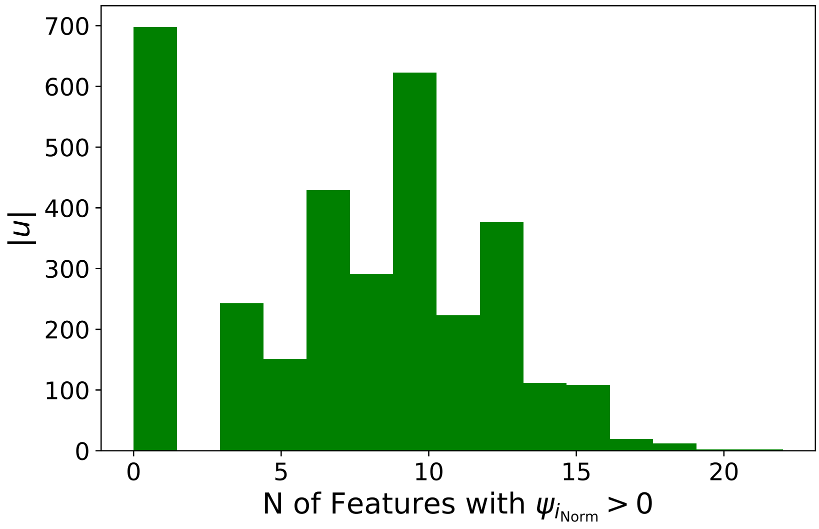

Figure S5: Count of non-zero $\psi_{i_{\text{Norm}}}[u]$ in each u - Iraq. The figure has been created using Matplotlib version 3.1.3. Url: https://matplotlib.org/3.1.3/contents.html.

**Weapon** x_j	**N of** $\psi_{j_{\text{Norm}}}[u] > 0$	**%**
Explosives	2476	75.28%
Firearms	1739	52.87%
Unknown.1	635	19.31%
Incendiary	107	3.25%
Melee	100	3.04%
Other	9	0.27%
Sabotage Equipment	5	0.15%

Table 5: Iraq Weapons - Number of non-zero $\psi_{j_{\text{Norm}}}[u]$ occurrences and percentage over U

Transportation, Unknown, Terrorists/Non-State Militia, Utilities, Military, Telecommunication, Educational Institution, Maritime, Tourists, Other, Food or Water Supply, Airports & Aircraft. The distribution of occurrences is displayed below (Table 6).

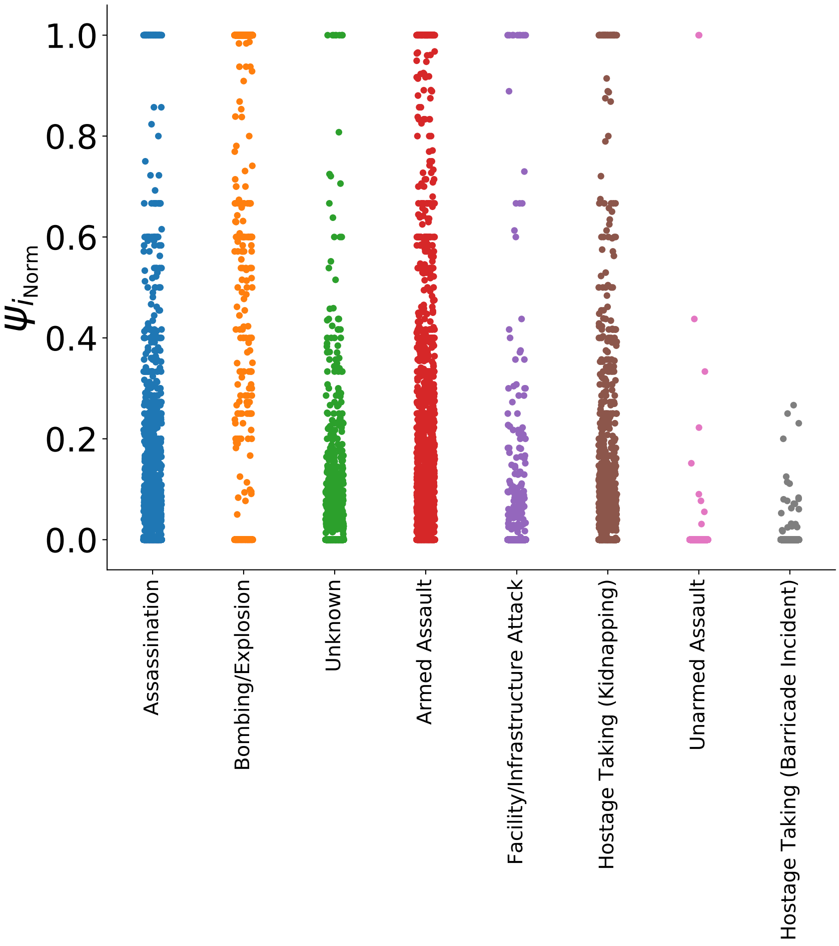

Figure S6: Distribution of $\psi_{i_{\text{Norm}}}[u]$ over U for all the tactics features X - Iraq. The figure has been created using Matplotlib version 3.1.3. Url: https://matplotlib.org/3.1.3/contents.html.

Given the low prevalence, *Maritime* has been excluded, leading to a total of 20 time series mapping targets in Iraq (Figure S8).

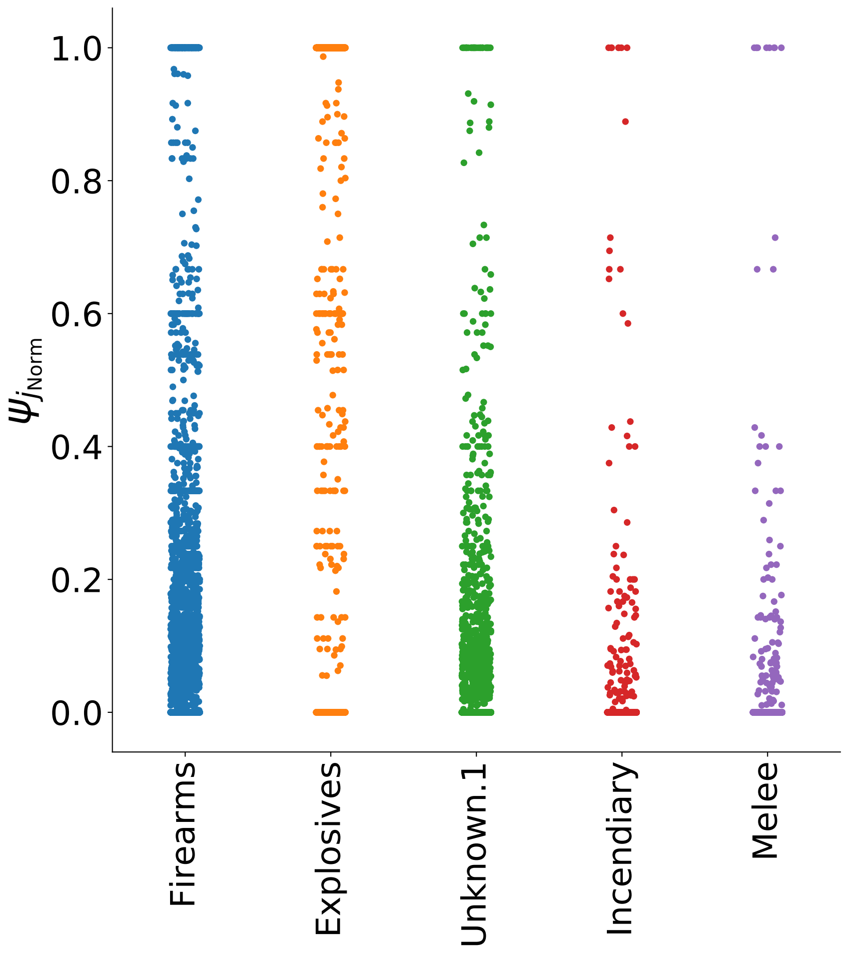

Figure S7: Distribution of $\psi_{j_{\text{Norm}}}[u]$ over U for all the weapon features W - Iraq. The figure has been created using Matplotlib version 3.1.3. Url: https://matplotlib.org/3.1.3/contents.html.

S2 Experiments

S2.1 Algorithms: Architectures' Details

This subsection provides details on the architectures of the different models, except the baseline which did not involve any learning mechanism. Overall, all models have been trained for 100 epochs using Adam as the optimizer given its ability in noisy problems involving sparse gradients (Kingma and Ba, 2017) and a batch size equal to 16. Furthermore, we set 10 as the patience hyper-parameter mapping the validation loss of the model. As described in the main manuscript, all models have been run using different input width in terms of time units u, to understand what the optimal length of the recent

**Target** y_k	**N of** $\psi_{k_{\text{Norm}}}[u] > 0$	**%**
Private Citizens & Property	2220	67.50%
Police	1602	48.71%
Government (General)	1326	40.32%
Business	1210	36.79%
Terrorists/Non-State Militia	747	22.71%
Military	599	18.21%
Unknown.2	566	17.21%
Religious Figures/Institutions	487	14.81%
Transportation	425	12.92%
Educational Institution	250	7.60%
Utilities	233	7.08%
Journalists & Media	168	5.11%
Government (Diplomatic)	105	3.19%
Violent Political Party	65	1.98%
Other.1	57	1.73%
Food or Water Supply	28	0.85%
Airports & Aircraft	27	0.82%
NGO	25	0.76%
Telecommunication	19	0.58%
Tourists	10	0.30%
Maritime	2	0.06%

Table 6: Iraq Targets - Number of non-zero $\psi_{k_{\text{Norm}}}[u]$ occurrences and percentage over U

history to take into account in order to obtain better forecasts is:[1] these input widths were 1 (=2 days), 5 (=10 days), 15 (=1 month), 30 (=2 months).

It will be noted that the architectures are not particularly complex, i.e., they do not involve multiple hidden layers in most cases: this is mostly due to the limited amount of data (in terms of u) at our disposal. We also performed experiments with more complex architectures made of a higher numbers of stacked layers (and higher number of units and filters), but the complexity of the networks did not lead to increments in algorithmic performance (while leading instead in higher computational costs). Nonetheless, the resulting outcomes of the models presented in the paper indicate that even simple learning

[1]The only exception is the Baseline model that, given its particular architecture, only used the previous time unit to infer forecasts at $u + 1$.

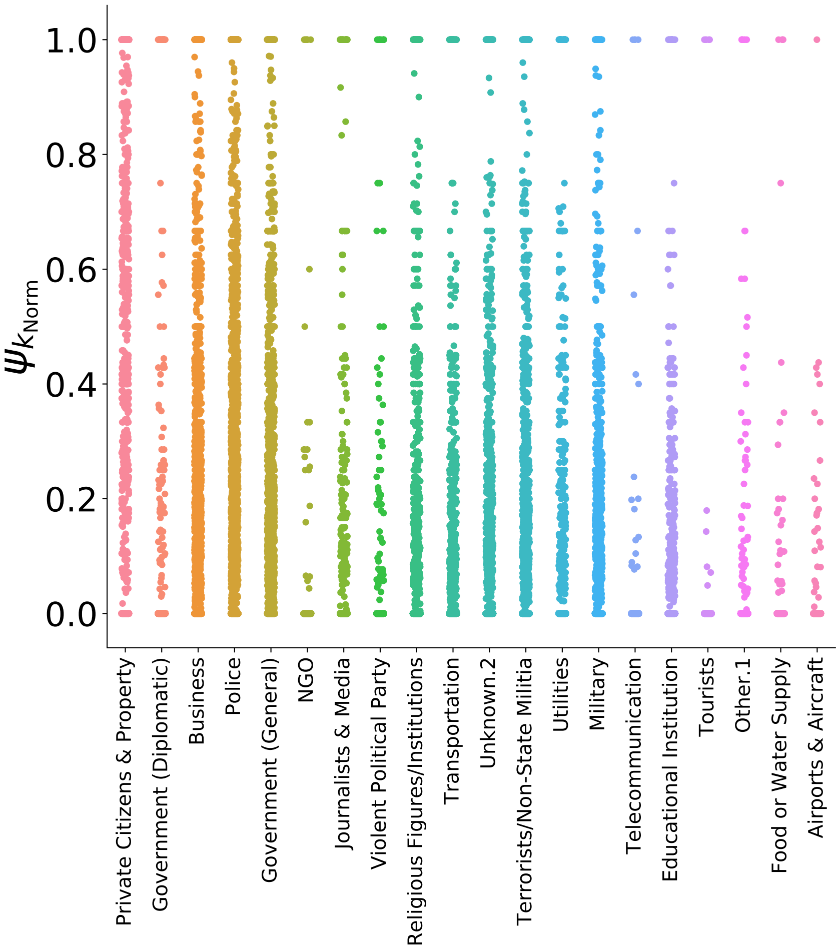

Figure S8: Distribution of $\psi_{k_{\mathrm{Norm}}}[u]$ over U for all the target features Y - Iraq. The figure has been created using Matplotlib version 3.1.3. Url: https://matplotlib.org/3.1.3/contents.html.

architectures are capable of efficiently forecasting terrorist targets. This aspect inserts in an emerging area of research that investigates the benefits of training simpler models over massive networks (Ba and Caruana, 2014). Instead of being a limitation, reaching good performance with simple and computationally cheap models may be considered a strength of the proposed computational framework and, particularly, of the engineering of our feature space. As a final note, in addition to the specific use of Dropout as a regularized for some models, all architectures have been trained using early stopping with a patience of 10 epochs in relation to Mean Squared Error to further limit the risk of overfitting.

S2.1.1 Feedforward Neural Network (FNN)

The FNNs trained in our experiments involved an input layer, followed by a flatten layer with 0 trainable parameters. Following, two dense layers with 32 neurons each with Rectified Linear Unit (ReLU) as the activation function. Finally, the last layer involved a number of units equal to the number of targets (i.e., 18 in the Afghanistan dataset, 20 in the Iraq one), and was followed by a reshape layer with no parameters. All dense layers had Glorot uniform as the kernel initializer and included a bias vector.

S2.1.2 Long Short-Term Memory Network (LSTM)

The LSTM networks involved a first input layer, followed by an LSTM layer with 32 neurons and a 0.5 dropout to avoid overfitting (Srivastava et al., 2014). The LSTM layer used Tanh as the activation function (given the need to forecast values in the $[0, 1]$ range) and Glorot uniform as the kernel initializer. The recurrent activation function was a sigmoid and the LSTM layer also included a bias vector. The recurrent initializer was an orthogonal matrix to prevent vanishing and exploding gradients (Le et al., 2015; Henaff et al., 2017). Finally, the last layer was a dense one having as units the number of targets in each dataset, and the same hyperparameters as the dense layers described in the FNN network.

S2.1.3 Convolutional Neural Network (CNN)

The trained CNNs have a total of four layers. Besides the first input one, we have included a 1D-Convolutional layer with 32 filters, followed by two dense layers (one with 32 units, the other with a number of units equal to $|Y|$), sharing the same hyperparameters of the dense layers in the previous models. The 1D-Convolutional layer made use of a bias vector with no padding. The activation function used in the 1D-Convolutional layer and the first dense layers was a ReLU.

S2.1.4 Bi-Directional Long Short-Term Memory Network (Bi-LSTM)

The Bi-LSTM networks are very similar in their architecture to the LSTM ones. The only (relevant) difference is that instead of having a simple LSTM layer, it actually has a bidirectional one (involving a double number of parameters), with concatenation as the merge mode. The dropout is set as 0.5. The Bidirectional layer uses Tanh as activation

and sigmoid as the recurrent activation function and allows the network to access the hidden state output at each input time unit.

S2.1.5 CNN-LSTM (CLDNN)

Finally, the CNN-LSTM model that takes inspiration from the CLDNN architecture proposed by Sainath et al. (2015), was engineered with a first input layer, followed by a 1D-Convolutional layer with 32 filters, a 1D-Max Pooling layer with pool size equal to 2, a dense layer with 32 units with ReLU as activation, an LSTM layer with 32 neurons, 0.5 dropout and Tanh as activation and a final dense layer with a number of units equal to Y. In the first layer (1D-Convolutional) there is no padding and Glorot uniform is used as the kernel initializer. In the 1D-Max Pooling layer also no padding is performed. The dense and LSTM remaining layers share the same hyperparameters of the previously outline dense and LSTM layers found in the other models.

S3 Additional Results

Figures S9 and S10 integrate the results presented in the main text, highlighting the correlation between each vector $\Psi_{norm}[u]$ and $\hat{\Psi}_{norm}[u]$, representing respectively the empirical centrality values of each target feature at each time stamp u and the corresponding predicted centrality values. Both graphs relates to the models reaching the highest Γ for the Afghanistan and Iraq cases.

While both correlation signals suffer from oscillations that, in certain cases, lead to unsatisfactory correlation values that get close to zero or become even negative, the mean correlation value for Afghanistan is 0.71 (with SD=0.23), while for Iraq is 0.69 (with SD=0.21). The first moment of both distributions corroborates the ability of the models with the highest performance in capturing the underlying dynamics and trend found in the empirical multivariate time-series.

Nonetheless, future work should investigate the reason of the negative oscillations to understand what causes them, while concurrently improve the overall forecasting performance at the general level (in terms of Γ and Φ) and at the target-level, to guarantee model interpretability and results validity, two core challenges in applied deep learning in many fields.

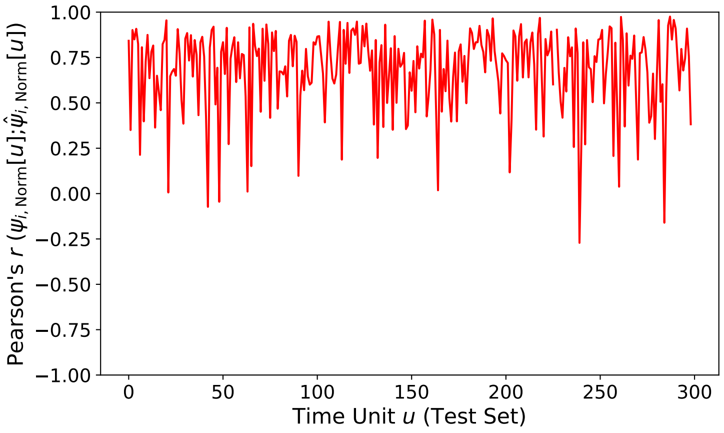

Figure S9: Correlation between empirical and forecasted centrality (test set) - Afghanistan. The figures has been created using Matplotlib version 3.1.3. Url: https://matplotlib.org/3.1.3/contents.html.

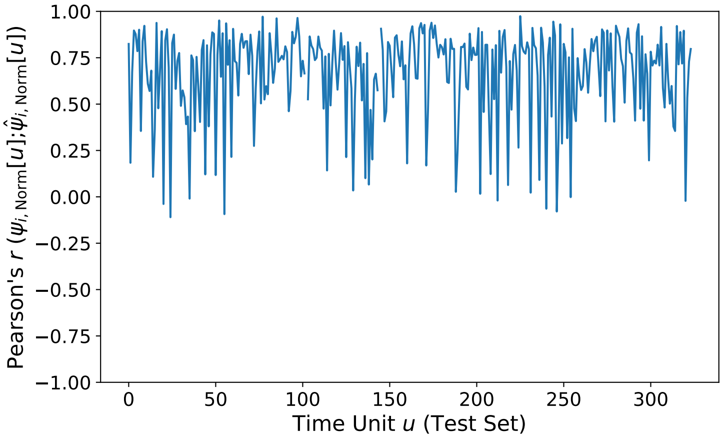

Figure S10: Correlation between empirical and forecasted centrality (test set) - Iraq. The figure has been created using Matplotlib version 3.1.3. Url: https://matplotlib.org/3.1.3/contents.html.

References

G. LaFree, L. Dugan, Introducing the Global Terrorism Database, Terrorism and Political Violence 19 (2007) 181–204. URL: http://dx.doi.org/10.1080/09546550701246817. doi:10.1080/09546550701246817.

D. P. Kingma, J. Ba, Adam: A Method for Stochastic Optimization, arXiv:1412.6980 [cs] (2017). URL: http://arxiv.org/abs/1412.6980, arXiv: 1412.6980.

J. Ba, R. Caruana, Do Deep Nets Really Need to be Deep?, in: Z. Ghahramani, M. Welling, C. Cortes, N. D. Lawrence, K. Q. Weinberger (Eds.), Advances in Neural Information Processing Systems 27, Curran Associates, Inc., 2014, pp. 2654–2662. URL: http://papers.nips.cc/paper/5484-do-deep-nets-really-need-to-be-deep.pdf.

N. Srivastava, G. Hinton, A. Krizhevsky, I. Sutskever, R. Salakhutdinov, Dropout: A Simple Way to Prevent Neural Networks from Overfitting, Journal of Machine Learning Research 15 (2014) 1929–1958. URL: http://jmlr.org/papers/v15/srivastava14a.html.

Q. V. Le, N. Jaitly, G. E. Hinton, A Simple Way to Initialize Recurrent Networks of Rectified Linear Units, arXiv:1504.00941 [cs] (2015). URL: http://arxiv.org/abs/1504.00941, arXiv: 1504.00941.

M. Henaff, A. Szlam, Y. LeCun, Recurrent Orthogonal Networks and Long-Memory Tasks, arXiv:1602.06662 [cs, stat] (2017). URL: http://arxiv.org/abs/1602.06662, arXiv: 1602.06662.

T. N. Sainath, O. Vinyals, A. Senior, H. Sak, Convolutional, Long Short-Term Memory, fully connected Deep Neural Networks, in: 2015 IEEE International Conference on Acoustics, Speech and Signal Processing (ICASSP), 2015, pp. 4580–4584. doi:10.1109/ICASSP.2015.7178838, iSSN: 2379-190X.
